# Breaking the Kinetics–Loading Trade‐Off in Zinc‐Ion Hybrid Capacitors Through Synergistic Electron–Ion Transport Design

**DOI:** 10.1002/advs.76823

**Published:** 2026-07-27

**Authors:** Jiacheng Wu, Yangyang Wang, Fangzhou Liu, Fangxin She, Justin Prabowo, Di Zhu, Xin Yang, Li Wei, Yuan Chen

**Affiliations:** ^1^ School of Chemical and Biomolecular Engineering The University of Sydney Darlington New South Wales Australia

**Keywords:** carbon nanotube, high‐mass‐loading electrode, ion transport kinetics, low‐tortuosity architecture, zinc‐ion hybrid capacitor

## Abstract

Zinc‐ion hybrid capacitors (ZIHCs) are promising for safe, low‐cost, large‐scale energy storage, yet the poor kinetics of high‐mass‐loading carbon cathodes hinder their practical deployment. Most reported ZIHCs employ low mass loadings (< 2 mg cm^−^^2^), resulting in overestimated electrochemical performance and limited practical relevance. Here, the kinetic limitations induced by high mass loading are systematically elucidated, and a synergistic transport‐engineering strategy is developed to overcome them. Increasing the mass loading from 2 to 10 mg cm^−^^2^ causes severe rate deterioration, with capacity retention decreasing from 43.8% to 17.0% at 20 A g^−^^1^, accompanied by increased charge‐transfer resistance and nearly one‐order‐of‐magnitude suppression of Zn^2^^+^ diffusion. To address these limitations, electronic and ionic transport are independently optimized through carbon nanotube (CNT) conductive networks and low‐tortuosity pore channels generated by directional freeze‐drying. While mesoporous carbons show limited benefits, aligned pore architectures accelerate ion transport, and CNTs effectively mitigate electronic resistance. Integrating these features enables simultaneous optimization of electron and ion pathways in thick electrodes. Consequently, a practical pouch cell with a high mass loading (10 mg cm^−^^2^) and low N/P ratio (∼ 16.4) delivers 38.3 mAh g^−^^1^ at 10 A g^−^^1^ over 3000 cycles. This work establishes a scalable electrode‐design strategy for practical high‐mass‐loading ZIHCs.

## Graphical abstract

High‐mass‐loading carbon cathodes are essential for practical zinc‐ion hybrid capacitors but suffer from severe transport limitations. By combining low‐tortuosity freeze‐dried pore channels with carbon nanotube conductive networks, simultaneous optimization of ion and electron transport is achieved, enabling high‐rate, long‐life pouch cells and revealing key design principles for practical thick electrodes.

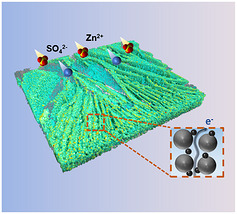

## 1 Introduction

The rapid expansion of electric vehicles, portable electronics, and grid‐scale energy storage has intensified demand for electrochemical energy storage systems that simultaneously deliver high energy and power densities, and long‐term cycling stability [1, 2, 3, 4]. Conventional metal‐ion batteries, such as lithium‐ion batteries, store energy via reversible Faradaic reactions but are limited by sluggish reaction kinetics and reduced capacity at high rates, which restricts their applicability in high‐power applications [5, 6, 7]. In contrast, electrochemical capacitors rely on interfacial ion adsorption/desorption processes, offering excellent power capability and cycling durability, albeit at the expense of low energy density [8, 9, 10]. Hybrid ion capacitors have therefore emerged as a potential solution by integrating a battery‐type anode with a capacitive cathode, achieving a favorable balance between energy density and power performance [11, 12, 13].

Among various hybrid systems, zinc‐ion hybrid capacitors (ZIHCs), typically consisting of a metallic Zn anode and a carbon‐based cathode, have attracted increasing attention. This interest stems from Zn's high theoretical capacity (823 mAh g^−^^1^), its low redox potential (−0.76 V vs. SHE), its intrinsic safety, and its low material cost, making ZIHCs particularly attractive for large‐scale energy storage applications [14, 15]. Carbon materials are widely regarded as ideal cathode materials for ZIHCs due to their high specific surface area, tunable porosity, chemical stability, and favorable pore structures for reversible Zn^2^^+^ adsorption/desorption [16, 17, 18]. In particular, micropores (< 2.0 nm) provide abundant adsorption sites for accommodating solvated [Zn(H_2_O)_6_]^2+^ ions (∼ 0.86 nm) in aqueous electrolytes, whereas larger mesopores (2–50 nm) can serve as electrolyte reservoirs and ion‐transport channels to promote efficient ion diffusion [19, 20]. Consequently, a range of carbon materials, including heteroatom‐doped porous carbons [21], carbon fibers [22], and hollow carbon spheres [23], have been developed as cathode materials for ZIHCs.

Despite significant progress in material design, a critical limitation remains: most reported ZIHCs employ cathodes with relatively low carbon material mass loadings, typically below 2 mg cm^−^^2^ [24, 25, 26, 27]. Such low mass loadings can lead to substantially overestimated rate capability and energy density, thereby limiting the practical relevance of these studies. Our recent work [28], together with several independent reports [29, 30, 31, 32], demonstrates that high‐mass‐loading carbon cathodes (> 10 mg cm^−^^2^) are essential for achieving ZIHCs with practical energy output. However, increasing mass loading inevitably introduces new challenges. Thick electrodes commonly exhibit elevated electronic resistance [33, 34] and increased pore tortuosity, which hinders electrolyte infiltration and ion transport, ultimately leading to deteriorated electrochemical kinetics and poor rate performance [35, 36].

Accordingly, the development of practical ZIHC cathodes requires a coordinated electrode design strategy that simultaneously reduces electronic resistance and promotes efficient ion transport. Several approaches have been proposed to address these challenges, including the construction of gradient or hierarchical pore structures [37], the fabrication of 3D‐printed electrodes [38], and the use of wood‐derived porous frameworks [30]. Nevertheless, a systematic investigation of how cathode mass loading influences electrochemical kinetics and performance in ZIHCs using commercial carbon materials remains lacking.

Herein, we first compare microporous carbon cathodes with two representative mass loadings (2 and 10 mg cm^−^^2^) in ZIHCs to elucidate the intrinsic effect of electrode thickness on electrochemical behavior. We then investigate three complementary strategies to improve the performance of high‐mass‐loading carbon cathodes: (i) incorporating carbon nanotubes (CNTs) as conductive additives to reduce electronic resistance; (ii) introducing mesoporous carbon materials with short‐range mesopores to optimize pore size distribution and ion‐accessible surface area; and (iii) constructing low‐tortuosity, vertically aligned long‐range pore channels via directional freeze‐drying. By correlating electrode structures, as characterized by scanning electron microscopy (SEM) and micro‐computed tomography (micro‐CT), with electrochemical performance, we show that long‐range pore channels, combined with low electronic resistance, are critical for achieving high‐rate capability and long‐term cycling stability. An optimized ZIHC pouch cell with a low negative‐to‐positive electrode capacity (N/P) ratio of 16.4 delivers a high specific capacity of 38.3 mAh g^−^^1^ at 10 A g^−1^ over 3000 charge–discharge cycles with a device‐level energy density of 2.82 Wh kg^−1^. This study provides useful insights into the relationship between cathode carbon material mass loading and electrochemical performance. It offers practical design guidelines for developing high‐mass‐loading carbon cathodes for ZIHCs.

## 2 Results and Discussion

### 2.1 Effect of Carbon Cathode Mass Loading

**FIGURE 1:**
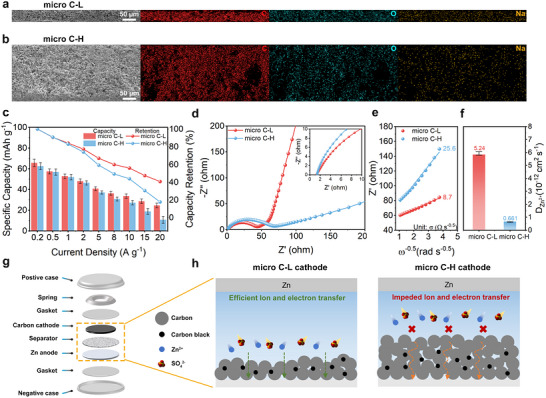
(a, b) Cross‐sectional SEM images and corresponding EDS mappings of C, O, and Na of micro C–L (2.0 mg cm^−2^) and micro C–H (10 mg cm^−2^) electrodes. (c) Specific capacity and rate retention of the carbon electrodes at different current densities (0.2–20 A g^−1^) tested using assembled coil cells. (d) Nyquist plots of the carbon electrodes (inset: enlarged Nyquist plots showing the R_s_) (e) The fitted linear relationship of Z’ and ω^−0.5^. (f) $D_{Zn^{2+}}$ in the carbon electrodes. (g) Schematic illustration of the structure of the CR2032 coin cell used to assemble ZIHCs in this study. (h) Schematic illustration of ion and electron transport in low‐ and high‐mass‐loading carbon electrodes in ZIHCs.

To elucidate the impact of increased mass loading on the electrochemical performance of carbon cathodes in ZIHCs, microporous carbon electrodes with two representative mass loadings, low (2.0 mg cm^−^^2^) and high (10 mg cm^−^^2^), were fabricated via the doctor‐blade coating method using microporous carbon (YP‐50F), a widely used commercial supercapacitor carbon. The electrodes are denoted as micro C–L and micro C–H, respectively. As shown in the cross‐sectional SEM images (Figure 1a,b), increasing the mass loading results in a fourfold increase in electrode thickness, from approximately 50 to ∼ 200 µm. Corresponding energy‐dispersive x‐ray spectroscopy (EDS) mappings reveal that the distributions of C, O, and Na become less uniform in the thick micro C–H electrode, indicating increased structural heterogeneity.

Figure 1c summarizes the gravimetric specific capacity and corresponding capacity retention of the two electrodes tested in assembled coin cells (see Supporting Information for device fabrication details) as the charge–discharge current density increases from 0.2 to 20 A g^−^^1^. The micro C–L electrode consistently delivers higher specific capacities across the entire current density range, achieving 65.3 mAh g^−^^1^ at 0.2 A g^−^^1^ and retaining 28.6 mAh g^−^^1^ at 20 A g^−^^1^, corresponding to 43.8% capacity retention. In contrast, the micro C–H electrode exhibits pronounced capacity decay, with the specific capacity decreasing from 61.1 mAh g^−^^1^ at 0.2 A g^−^^1^ to only 10.7 mAh g^−^^1^ at 20 A g^−^^1^, yielding a markedly lower capacity retention of 17.0%. While the performance difference between the two electrodes is relatively small at low current densities (0.2–2 A g^−^^1^), it becomes increasingly pronounced at higher current densities (5–20 A g^−^^1^), underscoring the detrimental effect of high mass loading on high‐rate performance.

Electrochemical impedance spectroscopy (EIS) was conducted to further investigate the underlying kinetic limitations. The Nyquist plots (Figure 1d) show that the micro C–L electrode exhibits a nearly vertical line in the low‐frequency range (0.01–1.0 Hz), approaching ideal capacitive behavior. In contrast, the micro C–H displays a pronounced semi‐infinite diffusion feature, indicative of diffusion‐controlled kinetics [39, 40]. Both electrodes exhibit comparable ohmic resistance (R_S_) values, 1.25 Ω for micro C–L and 1.38 Ω for micro C–H, suggesting similar bulk electrolyte and contact resistance [41]. However, the charge‐transfer resistance (R_ct_) increases substantially from 48.7 to 68.5 Ω with increasing mass loading, reflecting aggravated interfacial Faradaic kinetics. The Warburg coefficient (σ), extracted from the linear relationship between the real impedance (Z’) and the square root of angular frequency (ω^−^^1/2^) in the low‐frequency region, was used to evaluate ionic transport resistance [42]. As shown in Figure 1e, σ increases markedly from 8.7 to 25.2 Ω s^−^^1/2^ upon increasing the mass loading, corresponding to an almost one‐order‐of‐magnitude decrease in the Zn^2^^+^ diffusion coefficient ($D_{Zn^{2+}}$) from 5.24 × 10^−^^12^ to 0.661 × 10^−^^12^ cm^2^ s^−^^1^ (Figure 1f). This result highlights the severe ion transport limitation induced by the thick electrode architecture.

It should be noted that to isolate the influence of carbon material mass loading in cathodes, a CR2032 coin‐cell configuration was adopted in which all cell components were identical except for the carbon cathodes (Figure 1g). This thickness‐dependent behavior was further confirmed at the device level by comparing pouch cells using micro C–L and micro C–H cathodes (Figure S1), where the micro C–H pouch cell shows lower specific capacities and even worse capacity decay at high current densities. Collectively, these results indicate that the micro C–L electrode enables rapid and efficient transport of both electrons and ions, thereby sustaining high capacity retention at elevated current densities (Figure 1h). In contrast, the micro C–H electrode suffers from sluggish electronic and ionic transport, resulting in increased ohmic polarization and accelerated capacity fading during high‐rate operation [35, 36].

### 2.2 Incorporation of CNT Conductive Additives

**FIGURE 2:**
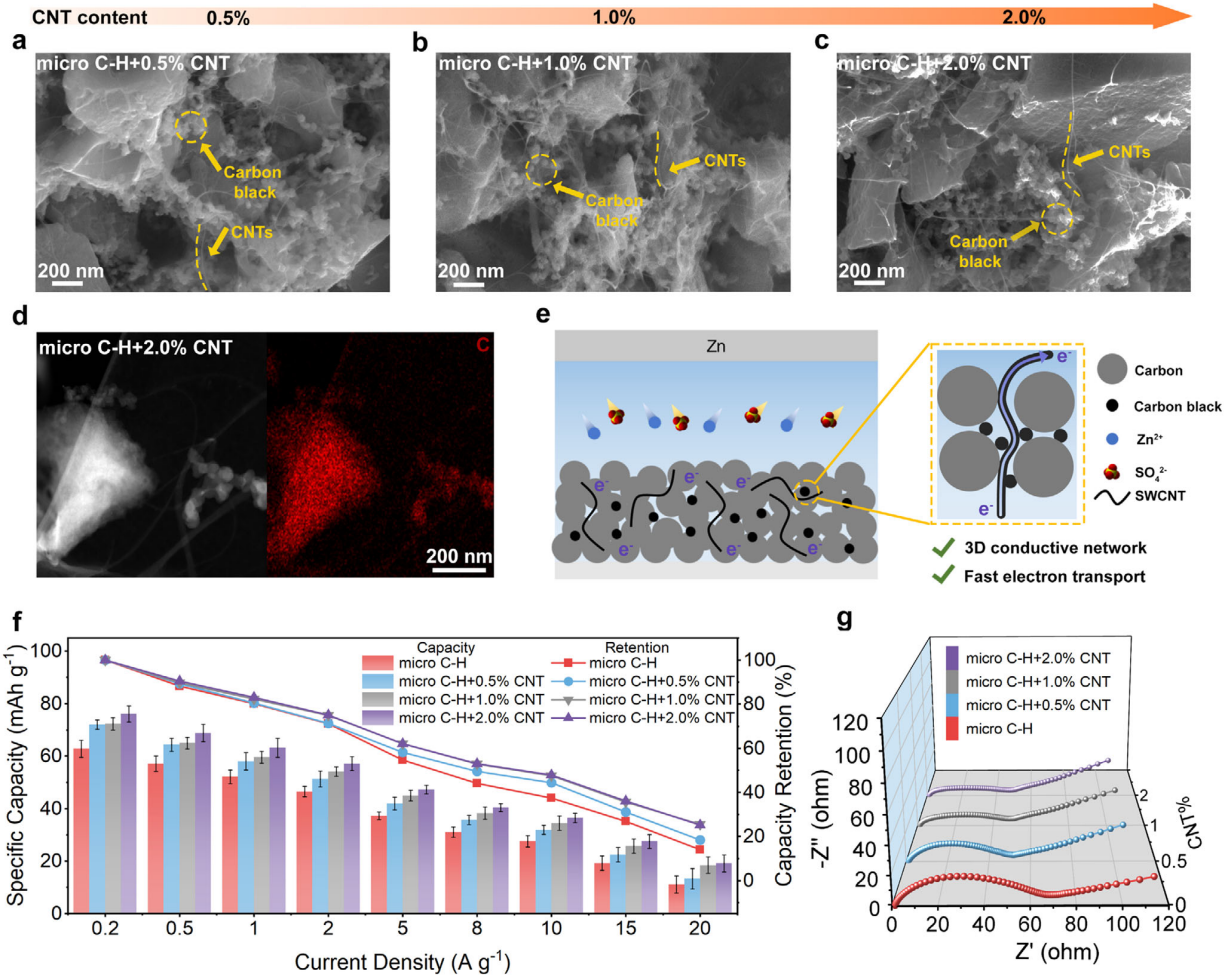
(a–c) SEM images of micro C–H+x% CNT electrodes, x% refers to 0.5, 1.0, or 2.0 wt.%, respectively. Typical CNTs and carbon black particles are highlighted in yellow. (d) HAADF‐STEM and element mapping images of micro C–H+2.0% CNT electrode. (e) Schematic illustration of enhanced electron transport in carbon electrodes with CNT conductive additives. (f) Specific capacity and rate retention of the carbon electrodes containing different amounts of CNTs. (g) Nyquist plots of micro C–H electrodes containing different amounts of CNTs.

Incorporating conductive additives is a widely adopted strategy to alleviate electronic transport limitations in thick electrodes. CNTs have previously been employed to improve the conductivity of high‐mass‐loading electrodes due to their high aspect ratio and excellent intrinsic electronic conductivity [43, 44, 45]. Here, commercial single‐walled CNTs (0.5–2.0 wt.%) were added into the microporous carbon (YP‐50F) cathodes with a fixed total mass loading of 10 mg cm^−^^2^ (denoted as micro C–H+x% CNT, x% refers to 0.5, 1.0, or 2.0 wt.%). SEM images (Figure 2a–c) reveal that long, entangled CNTs interconnect to form a continuous conductive network surrounding the spherical carbon particles, thereby improving particle–particle electrical contact. The HAADF‐STEM images (Figure 2d) further evidence the well distribution of carbon particles and CNT networks within the composite. As illustrated schematically in Figure 2e, CNTs bridge adjacent carbon particles to form an interconnected conductive framework, thereby reducing electronic resistance by generating new interparticle ionic transport pathways [46, 47, 48, 49]. As a result, both the specific capacity and rate capability of the electrodes are markedly enhanced (Figure 2f). Compared with the pristine microporous carbon electrode (micro C–H), all CNT‐containing electrodes exhibit improved performance, with the micro C–H+2.0% CNT electrode delivering the best results, followed by the micro C–H+1.0% CNT and micro C–H+0.5% CNT electrodes. Specifically, the micro C–H+2.0% CNT electrode achieves a high specific capacity of 76.1 mAh g^−^^1^ at 0.2 A g^−^^1^ and retains 19.1 mAh g^−^^1^ at 20 A g^−^^1^. Both micro C–H+1.0% CNT and micro C–H+2.0% CNT electrodes exhibit approximately 25% capacity retention at 20 A g^−^^1^, significantly outperforming the pristine micro C–H electrode. EIS further confirms the improvement in electronic conductivity (Figure 2g). R_ct_ (Table S1) decreases progressively from 68.5 Ω for the micro C–H electrode to 43.7 Ω (micro C–H+0.5% CNT), 36.6 Ω (micro C–H+1.0% CNT), and 30.7 Ω (micro C–H+2.0% CNT) with increasing CNT content. Similarly, the extracted σ values (Figure S2a) decrease from 25.6 Ω S^−0.5^ of micro C–H to 18.8, 18.1, and 19.6 Ω S^−0.5^ of the 0.5%, 1.0%. and 2.0% CNT electrodes, respectively. Thus, $D_{Zn^{2+}}$ (Figure S2b) is enhanced to 1.25, 1.37, and 1.26 × 10^−12^ cm^2^ s^−1^, confirming the CNT incorporation improves both charge‐transfer and Zn^2+^ diffusion.

The long‐term cycling stability of the assembled coin cells was evaluated at a high current density of 10 A g^−^^1^ (Figure S3). The ZIHC with the micro C–H+2.0% CNT electrode shows stable cycling over 10,000 cycles, delivering a specific capacity of 36.8 mAh g^−^^1^ with a high Coulombic efficiency of 99.8%. In contrast, the ZIHC assembled with the micro C–H electrode retains only 26.7 mAh g^−^^1^ under the same conditions. These results demonstrate that CNT incorporation mitigates electronic transport limitations to some extent in high‐mass‐loading carbon cathodes, thereby enhancing both rate capability and long‐term stability.

### 2.3 Incorporating Short‐Range Mesopores

Mesoporous carbons with short‐range mesopore networks have been reported to facilitate ionic diffusion in carbon electrodes [15, 50, 51, 52]. To evaluate their effectiveness in high‐mass‐loading ZIHC cathodes, two commercial mesoporous carbons with distinct pore characteristics (HPC10 and HPC12) were blended with microporous YP‐50F to fabricate composite electrodes.

**FIGURE 3:**
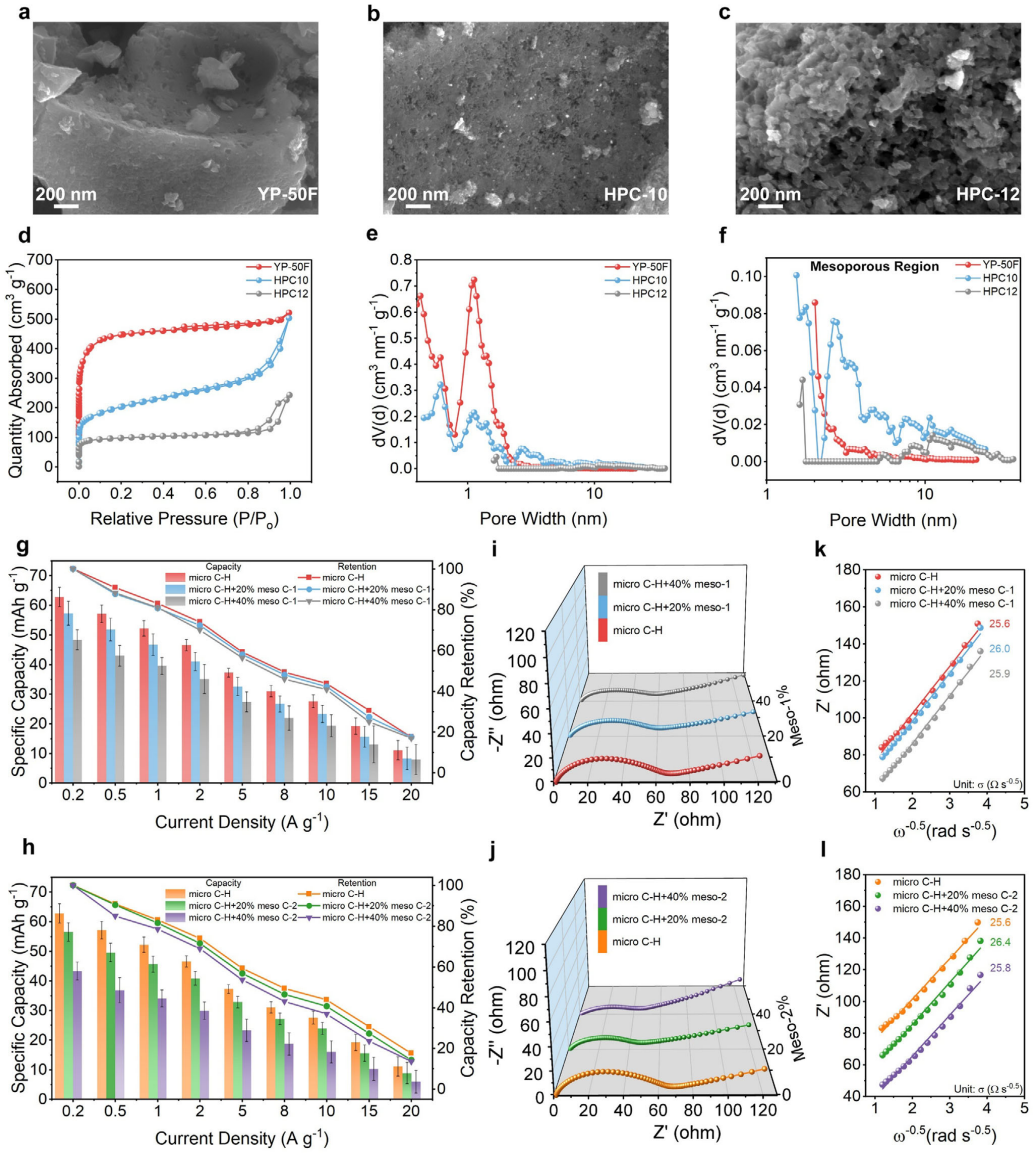
(a–c) SEM images of microporous and mesoporous carbons: (a) YP‐50F, (b) HPC10, (c) HPC12. (d) Nitrogen physisorption isotherms, (e) pore size distribution, and (f) DFT‐model‐based mesopore size distribution of YP‐50F, HPC10, and HPC12. (g, h) Specific capacity and rate retention of carbon composite electrodes blended with different mesoporous carbons (g) micro C–H+x% meso C‐1 electrodes (x refers to mass fraction of HPC10 in the electrode at 20, 40 wt.%) and (h) micro C–H+x% meso C‐2 electrodes (x refers to mass fraction of HPC12 in the electrode at 20, 40 wt.%). (i, j) The corresponding Nyquist plots of (i) micro C–H+x% meso C‐1 and (j) micro C–H+x% meso C‐2 electrodes. (k, l) The fitted linear relationship of Z’ and ω^−0.5^ for (k) micro C–H+x% meso C‐1 and (l) micro C–H+x% meso C‐2 electrodes.

SEM images (Figure 3a–c and Figure S4) reveal distinct surface morphologies among the three carbons. YP‐50F exhibits a relatively smooth granular surface composed of densely packed carbon fragments. In contrast, HPC10 shows compact carbon aggregates with small, distributed voids, while HPC12 presents similar agglomerated structures with larger interparticle features. XRD patterns (Figure S5) display broad diffraction peaks at ∼23° and ∼43°, corresponding to the (002) and (100)/(101) planes of graphitic carbon, indicating predominantly amorphous structures in all samples [53, 54, 55]. Raman spectra (Figure S6) show I_D_/I_G_ ratios of 1.12 (YP‐50F), 1.01 (HPC10), and 0.89 (HPC12), suggesting progressively higher graphitization from YP‐50F to HPC12 [21, 23]. XPS analysis (Figures S7–S9 and Table S2) confirms similar surface C and O compositions among the three carbons [56, 57, 58, 59]. N_2_ physisorption measurements were performed to determine the specific surface area (SSA) and pore size distribution. All samples exhibit type IV isotherms with H4 hysteresis loops (Figure 3d), indicating the coexistence of micro‐ and mesopores [41, 60, 61, 62]. YP‐50F shows a sharp adsorption increase at low relative pressures (P/P_0_ < 0.1), confirming its micropore‐dominated structure. HPC10 displays a pronounced hysteresis loop, consistent with well‐developed mesoporosity, while HPC12 shows lower adsorption capacity with a steep rise near P/P_0_ ≈ 1.0, indicating the presence of large meso‐ and macropores [63, 64, 65, 66, 67]. Pore size analysis by the DFT method (Figure 3e,f) reveals that YP‐50F is dominated by micropores (< 2 nm), whereas HPC10 contains dual micropore peaks (∼ 0.79 and ∼ 1.13 nm) along with a broad mesopore distribution (2–10 nm). HPC12 features larger mesopores ranging from 5 to 35 nm. As summarized in Table 1, YP‐50F has the highest SSA (S_BET_ = 1708 m^2^ g^−^^1^), with 80.4% of its pore volume contributed by micropores. HPC10 has a lower SSA (729 m^2^ g^−^^1^) but a predominantly mesoporous volume (75.9%). HPC12 exhibits the lowest SSA (373.9 m^2^ g^−^^1^) and total pore volume (0.376 cm^3^ g^−^^1^). Based on these structural characteristics, YP‐50F is expected to provide abundant microporous sites for charge storage, whereas HPC10 and HPC12 may facilitate ion transport through mesoporous pathways.

**TABLE 1:** The specific surface area and pore structures of YP‐50F, HPC10, and HPC12.

Sample	S_BET_ (m^2^ g^−1^)	V_total_ (cm^3^ g^−1^)	V_micro_ (cm^3^ g^−1^)	V_meso_ (cm^3^ g^−1^)	V_micro_/V_total_ (%)	V_meso_/V_total_ (%)	D_ave_ (nm)
**YP‐50F**	1708.2	0.807	0.649	0.158	80.4	19.6	1.89
**HCP‐10**	729.3	0.779	0.188	0.591	24.1	75.9	4.27
**HCP‐12**	373.9	0.376	0.133	0.243	35.3	64.6	4.02

To examine this hypothesis, HPC10 or HPC12 was blended with YP‐50F at mass ratios of 20, 40 wt.% to fabricate high‐mass‐loading (10 mg cm^−^^2^) composite electrodes (denoted as micro C–H+x % meso C‐1 and micro C–H+x% meso C‐2, where x refers to the mass fraction of HPC10 (meso C‐1) or HPC12 (meso C‐2) in the electrode. As shown in Figure 3g, specific capacity decreases with increasing current density (0.2–20 A g^−^^1^) for all the electrodes. The pristine micro C–H electrode delivers the highest specific capacity across all current densities. Incorporation of HPC10 results in a progressive decline in specific capacity, with the micro C–H+40% meso C‐1 electrode exhibiting 48.4 mAh g^−^^1^ at 0.2 A g^−^^1^ and 19.3 mAh g^−^^1^ at 10 A g^−^^1^. Moreover, the capacity retention of the HPC10‐containing electrodes (Figure 3g) remains comparable to that of the pristine micro C–H electrode, suggesting that HPC10 incorporation does not improve capacity retention in thick electrodes. EIS analysis (Figure 3i and Table S3) shows that R_ct_ decreases from 68.5 Ω (micro C–H) to 49.5 Ω (40% meso C‐1), reflecting improved electronic conductivity due to the higher graphitization level of HPC10 [68, 69, 70]. However, the σ (Figure 3k) and calculated $D_{Zn^{2+}}$ (Figure S10a) remain close (6.61 × 10^−^^13^ to 6.46 × 10^−^^13^ cm^2^ s^−^^1^), indicating negligible improvement in ionic transport.

A similar decreasing capacity trend is observed for HPC12‐containing electrodes (Figure 3h). For instance, the micro C–H+40% meso C‐2 electrode delivers only 16.0 mAh g^−^^1^ at 10.0 A g^−1^. Meanwhile, the capacity retention of the HPC12‐containing electrodes (Figure 3h) shows a slight decrease compared with that of the pristine micro C–H electrode. Although R_ct_ (Figure 3j and Table S3) decreases further (down to 32.6 Ω) for micro C–H+40% meso C‐2, σ (Figure 3l) and $D_{Zn^{2+}}$ (Figure S10b) show no significant enhancement.

To quantitatively assess the structural characteristics, synchrotron‐based micro‐CT analysis was performed. As shown in Figure S11, the overall 2D cross‐sectional micro‐CT slices yield similar 3D volume renderings for both meso electrodes. The corresponding porosity (ɛ) and tortuosity (τ) analysis reveals comparable results, with ɛ values of 0.10 and 0.14, and τ values of 3.11 and 2.61 for micro C–H+40% meso C‐1 and micro C–H+40% meso C‐2, respectively (Figure S12 b,c,f,g and Table S4). Nevertheless, it should be noted that micro‐CT's spatial resolution is limited to the micrometer scale, hindering its ability to resolve mesopores and macropores effectively. Despite this, the comparable macroscopic tortuosity further supports that these short‐range mesopores do not create effective long‐range ion‐transport pathways in thick electrodes.

Overall, these results demonstrate that incorporating mesoporous carbons (HPC10 or HPC12) into high‐mass‐loading electrodes does not improve either specific capacity or capacity retention. Two coupled factors account for this behavior. First, the substantially lower SSA of HPC10 and HPC12 reduces the overall accessible surface area when partially replacing YP‐50F, directly decreasing charge storage capacity [71, 72, 73, 74]. Second, although these mesoporous carbons introduce short‐range mesopores and improve electronic conductivity to some extent, the nearly unchanged capacity retention, $D_{Zn^{2+}}$, and macroscopic tortuosity indicate that short‐range mesopores do not effectively alleviate ionic transport limitations, especially in thick electrodes, and persistent tortuosity constraints remain [75, 76]. These findings highlight that simply introducing short‐range mesopores at the expense of surface area is insufficient; instead, constructing long‐range, low‐tortuosity ionic transport pathways while maintaining high surface area is essential for optimizing high‐mass‐loading electrodes [77, 78].

### 2.4 Forming Long‐Range Pore Channels

To overcome the ionic transport limitations identified above, we introduced long‐range pore channels into high‐mass‐loading electrodes via a directional freeze‐drying strategy. A previously reported ice‐templating method was employed using the water‐based CMC binder [35, 79]. As illustrated in Figure S13a, a homogeneous electrode slurry (10 mg cm^−^^2^) was first coated onto Ti foil to form a uniform film, which was then placed on a Cu plate (Figure S13b). The bottom of the Cu plate was then immersed directly into liquid nitrogen, generating a steep temperature gradient that induced the directional growth of ice crystals within the slurry (Figure S13c). After solidification, the frozen film was freeze‐dried at −60°C under 0.01 mbar overnight to sublimate the ice crystals, yielding dried carbon electrodes (denoted as DFD‐micro C–H). Such a directional freeze‐drying strategy shows potential scalability toward high‐mass‐loading electrodes (i.e., > 10 mg cm^−2^) by tuning the coating thickness. It helps mitigate cracking issues commonly encountered in conventionally dried thick electrodes [80, 81].

**FIGURE 4:**
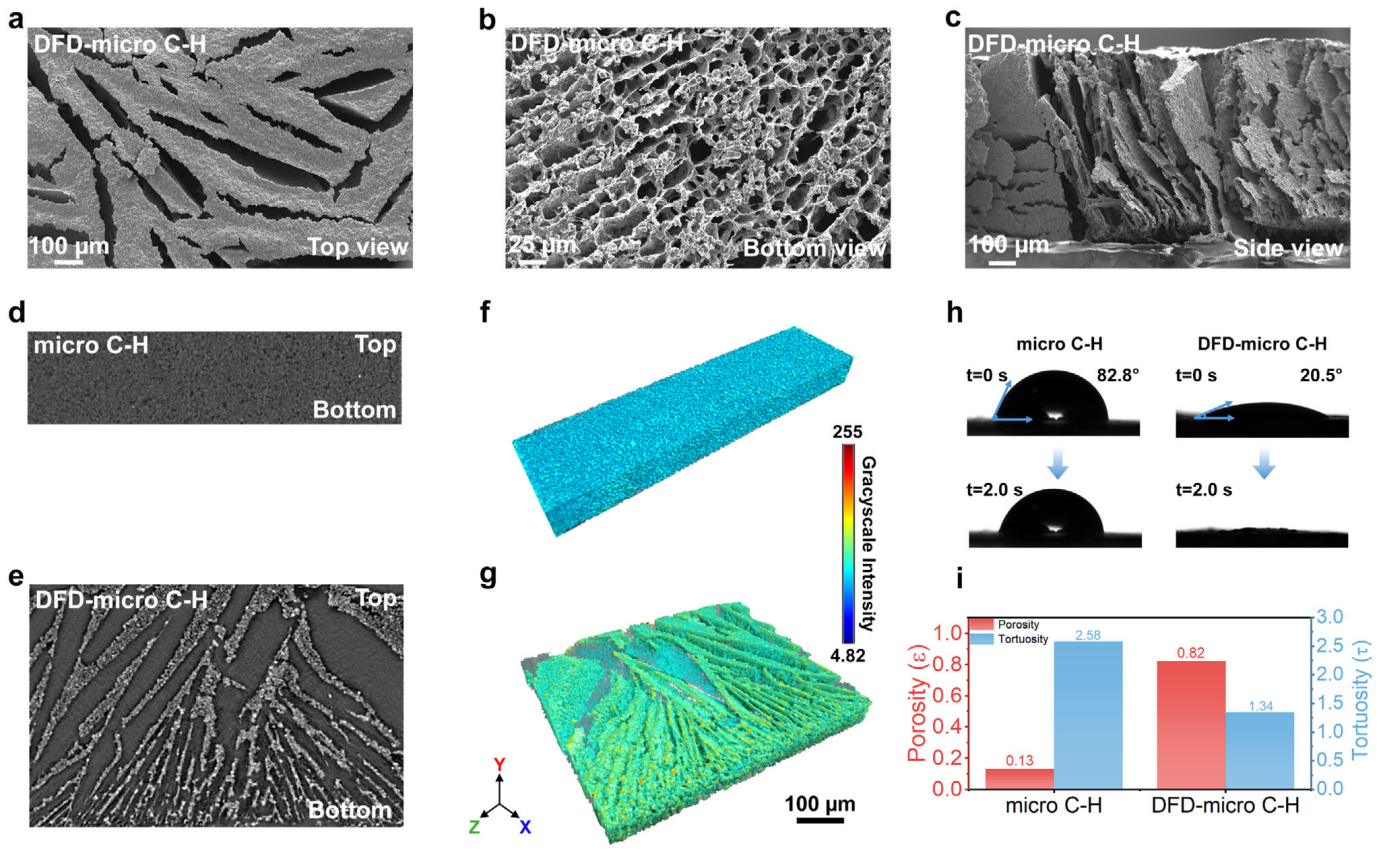
(a–c) SEM images of top, bottom, and side views of DFD‐micro C–H electrodes. (d‐e) 2D cross‐sectional micro‐CT slices of the (d) micro C–H and (e) DFD‐micro C–H electrodes. (f, g) 3D micro‐CT volume rendering of the reconstructed (f) micro C–H and (g) DFD‐micro C–H electrodes. (h) Surface contact angle measurements of micro C–H and DFD‐micro electrodes. (i) Porosity and tortuosity of micro C–H and DFD‐micro C–H electrodes.

SEM characterization reveals significant structural differences between the DFD‐micro C–H and micro C–H electrodes. The top surface of the DFD‐micro C–H electrode (away from the Cu substrate) exhibits numerous open and continuous pore channels (Figure 4a). In contrast, the bottom surface (near the Cu substrate) displays an interconnected honeycomb‐like network with pore sizes of approximately 10–20 µm (Figure 4b). Cross‐sectional images (Figure 4c) confirm the presence of vertically aligned channels extending throughout the electrode thickness. Notably, the electrode thickness increases from ∼ 200 µm (micro C–H) to ∼ 600 µm (DFD‐micro C–H), indicating substantial pore formation resulting from ice templating [79, 82]. Despite the increased thickness, the DFD‐micro C–H electrode can be readily compressed during cell assembly, resulting in a working thickness of ∼ 330 µm, which is comparable to that of the dense micro C–H electrode (Figure S14).

The 2D cross‐sectional images and 3D volume rendering show that the micro C–H electrode (Figure 4d,f) consists of densely packed carbon particles with highly tortuous ion transport pathways. In contrast, the DFD‐micro C–H electrode (Figure 4e,g) exhibits an anisotropic, low‐tortuosity architecture featuring continuous long‐range pore channels. Consistent with SEM observations, vertically aligned channels are evident near the top region, while lamellar‐like channels appear near the bottom region. These hierarchical channels are expected to facilitate rapid electrolyte infiltration and efficient ion transport throughout the thick electrode.

Wettability measurements (Figure 4h) further support this interpretation. The DFD‐micro C–H electrode shows a significantly reduced contact angle (28.5°) compared to the micro C–H electrode (82.8°), indicating enhanced electrolyte affinity, as well as rapid electrolyte infiltration time. Porosity and tortuosity analysis (Figure 4i, Figure S12d,h, and Table S4) reveals that the DFD‐micro C–H electrode possesses substantially higher porosity (ε = 0.83) than the micro C–H electrode (ε = 0.13). In contrast, its tortuosity is markedly reduced (τ = 1.34 vs. 2.58), demonstrating improved pore connectivity and shortened ion transport pathways. The synergistic increase in porosity and decrease in tortuosity directly promote the effective ionic diffusivity (*D_eff_*), which can be described by *D_eff_* =  (ε/τ) × *D*_0_, where *D*_0_ is the intrinsic Zn^2+^ diffusivity in 2 m ZnSO_4_ electrolyte. Therefore, the high ε/τ ratio of the DFD‐micro C–H electrode enables faster Zn^2+^ transport throughout the electrode [83].

**FIGURE 5:**
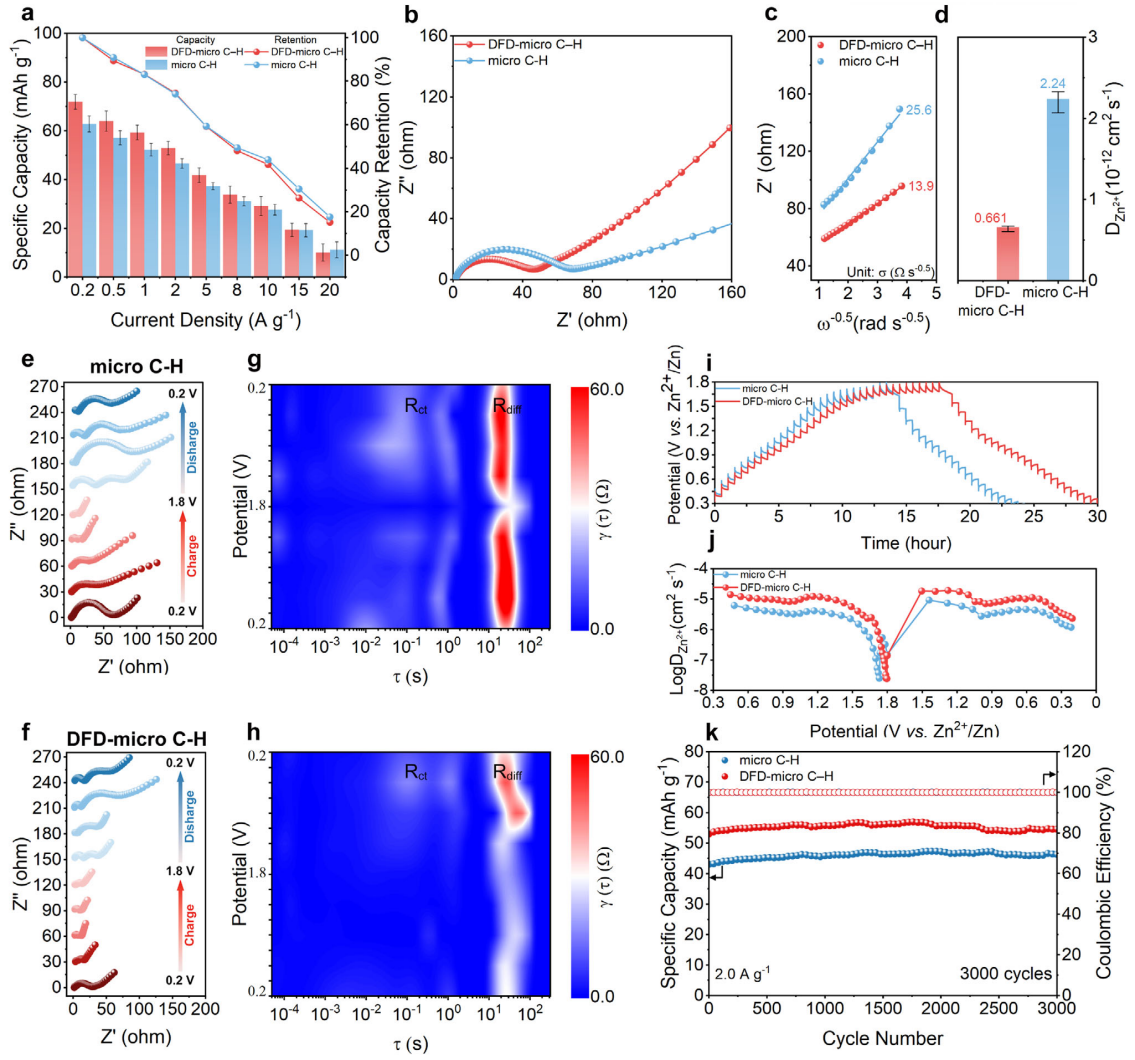
(a) Specific capacity and rate retention of micro C–H electrodes and DFD‐micro C–H electrodes. (b) Nyquist plots of DFD‐micro C–H and micro C–H electrodes. (c) The fitted linear relationship of Z’ and ω^−0.5^ for DFD‐micro C–H and micro C–H electrodes. (d) $D_{Zn^{2+}}$ of DFD‐micro C–H and micro C–H electrodes. (e, f) in situ EIS plots of (e) micro C–H and (f) DFD‐micro C–H electrodes. (g, h) DRT spectra contour plots of (g) micro C–H and (h) DFD‐micro C–H electrodes. (i) GITT curves of micro C–H and DFD‐micro C–H electrodes. (j) Log ($D_{Zn^{2+}}$) values of micro C–H and DFD‐micro C–H electrodes. (k) Long‐term cycling stability of micro C–H and DFD‐micro C–H electrodes at 2.0 A g^−1^.

The introduction of long‐range channels results in significant improvements in electrochemical performance. As shown in Figure 5a, the DFD‐micro C–H electrode delivers higher specific capacities than the micro C–H electrode at low‐to‐moderate current densities, achieving 71.4 mAh g^−^^1^ at 0.2 A g^−^^1^. However, this advantage diminishes at higher current densities and reverses at 15–20 A g^−^^1^, suggesting that electronic conductivity may become the limiting factor at high rates. EIS analysis (Figure 5b) shows that R_ct_ decreases from 68.5 Ω (micro C–H) to 49.6 Ω (DFD‐micro C–H). Meanwhile, σ is reduced to 13.9 Ω s^−^^0.5^ (Figure 5c), corresponding to an increased $D_{Zn^{2+}}$ to 2.24 × 10^−^^12^ cm^2^ s^−^^1^ (Figure 5d). To further explore the impedance evolution, an in situ EIS electrochemical test was performed during a full charge/discharge cycle. As shown in Figure 5e,f, the charge transfer resistance of micro C–H continues to show a much greater impedance circle across the full cycle. Additionally, the distribution of relaxation time (DRT) analysis was deconvoluted into contour maps (Figure 5g,h and Figure S15) to decouple different electrochemical processes. Notably, within the low‐frequency region (i.e., τ = 10–100 s), which is associated with the bulk diffusion of Zn^2+^ ions, the DFD‐micro C–H electrode displays significantly lower DRT signal intensity, confirming a significantly reduced ion diffusion resistance (R_diff_) at the electrode/electrolyte interface [84, 85]. Galvanostatic intermittent titration technique (GITT) measurements (Figure 5i) were conducted further to verify the rapid diffusion kinetics of DFD‐micro C–H. Accordingly, as shown in Figure 5j, log($D_{Zn^{2+}}$) of DFD‐micro C–H is determined to range from −4.85 to −7.61 cm^2^ s^−1^, surpassing the micro C–H (−5.2 to −7.60 cm^2^ s^−1^). As a result, DFD‐micro C–H maintains a high capacity of 56.2 mAh g^−1^ at 2.0 A g^−1^, along with a nearly 100% capacity retention over 3000 charge/discharge cycles at 2.0 A g^−1^ (Figure 5k).

### 2.5 Synergistic Effects of CNT Additives and Long‐Range Pore Channels

Finally, we integrated CNT conductive additives with the long‐range pore channels generated by directional freeze‐drying to simultaneously optimize electronic and ionic transport. The resulting electrodes are denoted as DFD‐micro C–H+x% CNT electrodes (x refers to CNT mass loadings of 0.5, 1.0, and 2.0 wt.%). Their electrochemical performance was benchmarked against the pristine DFD‐micro C–H electrode without CNTs.

**FIGURE 6:**
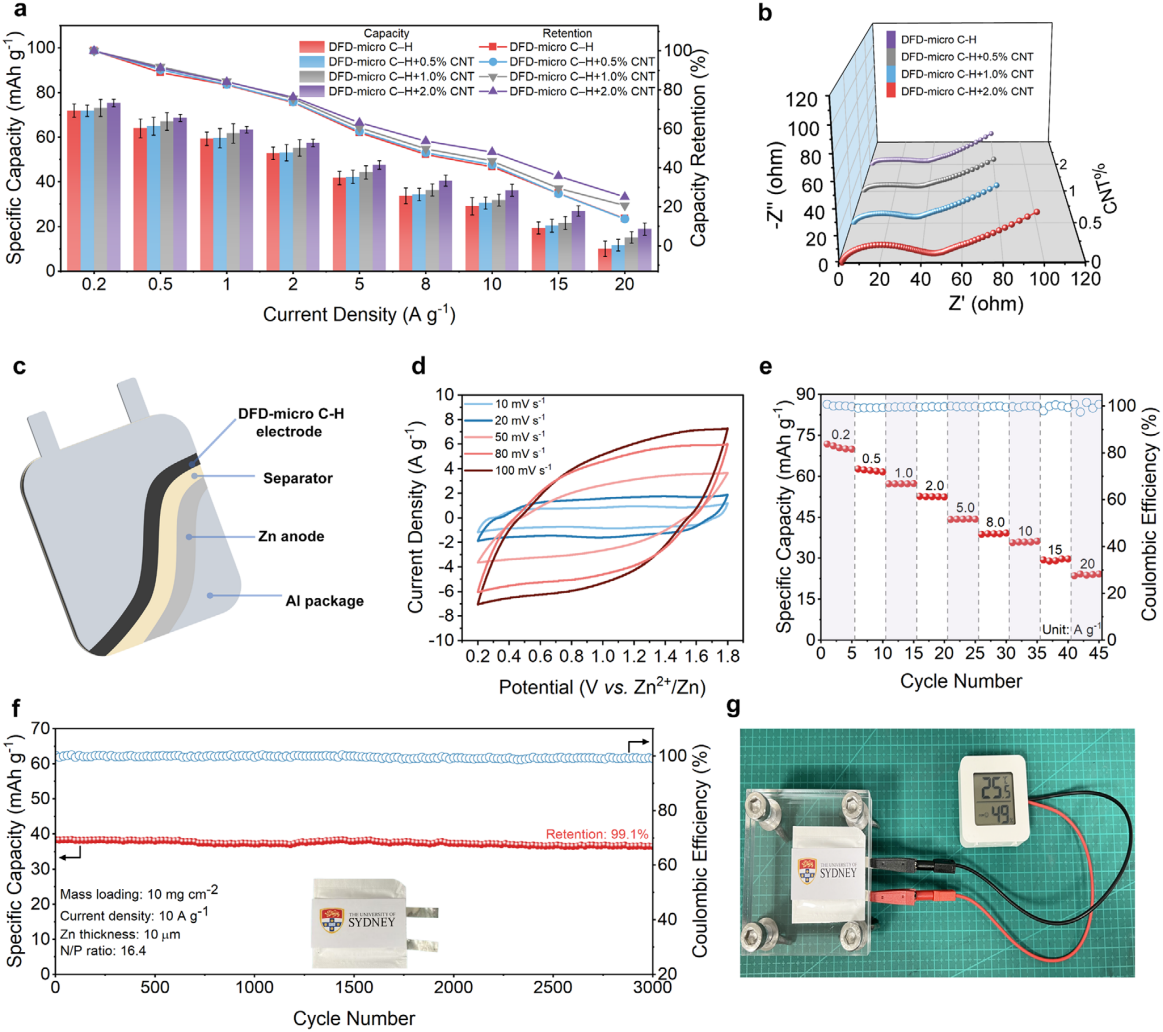
(a) Specific capacity and rate retention of DFD‐micro C–H+x% CNT electrodes (x refers to CNT mass loadings of 0.5, 1.0, 2.0 wt.%). (b) Nyquist plots of DFD‐micro C–H electrodes containing different amounts of CNTs. (c) Schematic illustration of the assembled DFD‐micro C–H+2.0% CNT pouch cell. (d) CV curves of Zn||DFD‐micro C–H+2.0% CNT pouch cell at different scan rates (10–100 mV s^−1^). (e) Rate performance at various current densities (0.2–20 A g^−1^). (f) Long‐term cycling stability of Zn||DFD‐micro C–H+2.0% CNT pouch cell at 10.0 A g^−1^. (g) A photo of a thermohydrometer powered by the Zn||DFD‐micro C–H+2.0% CNT pouch cell.

SEM images (Figure S16) confirm that incorporation of CNTs does not disrupt the vertically aligned pore architecture formed by directional freeze‐drying, indicating that the structural advantages of the DFD‐micro C–H electrode are preserved. As shown in Figure 6a, all DFD‐micro C–H+x% CNT electrodes exhibit higher specific capacities and improved rate retention compared to the DFD‐micro C–H electrode. A progressive enhancement in high‐rate performance is observed with increasing CNT content. Among them, the DFD‐micro C–H+2.0% CNT electrode delivers the best rate capability, retaining 18.9 mAh g^−^^1^ at 20 A g^−^^1^. EIS analysis (Figure 6b and Table S5) reveals a further decrease in R_ct_ upon CNT incorporation, confirming enhanced electronic conductivity. In contrast, the σ and $D_{Zn^{2+}}$ (Figure S17) remain comparable among the DFD micro C–H+x% CNT electrodes, suggesting that ion transport enhancement has already reached an optimized level due to the long‐range pore channels. These results demonstrate that, while the directional freeze‐drying strategy effectively improves ionic transport, CNT additives play a critical, complementary role in mitigating electronic transport limitations, thereby enabling superior high‐rate performance.

To further assess practical applicability, a pouch cell was assembled using a thin Zn foil (10 µm), as illustrated in Figure 6c. The cyclic voltammetry (CV) curves of the pouch cell (Figure 6d), recorded over a voltage window of 0.2–1.8 V at scan rates from 10 to 100 mV s^−^^1^, exhibit symmetric quasi‐rectangular shapes at low scan rates (10–50 mV s^−^^1^), indicating dominant capacitive behavior. Slight distortions appear at higher scan rates (80–100 mV s^−^^1^), reflecting increasing polarization [24]. The rate performance of the pouch cell (Figure 6e) shows that the specific capacity decreases gradually from 70.0 to 24.3 mAh g^−^^1^ as the current density increases from 0.2 to 20 A g^−^^1^, demonstrating robust rate capability. Long‐term cycling stability was evaluated at 10 A g^−^^1^ (Figure 6f). Notably, the pouch cell maintains a specific capacity of 38.3 mAh g^−^^1^ over 3000 cycles under a practical N/P ratio of ∼ 16.4, corresponding to a Coulombic efficiency of 99.1%. Additionally, a practical device‐level energy density of 2.82 Wh kg^−1^ based on a total device weight of 0.746 g (Table S6) was achieved. Finally, a fully charged Zn||DFD‐micro C–H + 2.0% CNT pouch cell successfully powered a thermohydrometer (Figure 6g), demonstrating its practical feasibility.

## 3 Conclusion

In summary, we systematically elucidated how increasing carbon cathode mass loading affects the electrochemical kinetics of ZIHCs and established a synergistic strategy to overcome the resulting transport limitations. Increasing the cathode mass loading from 2 to 10 mg cm^−2^ caused severe rate deterioration, with capacity retention decreasing from 43.8% to 17.0% at 20 A g^−1^ due to aggravated electronic and ionic transport limitations. A systematic comparison of three engineering strategies revealed that CNT conductive additives effectively improve electronic transport, whereas introducing short‐range mesoporous carbons provides negligible benefit for thick electrodes because the reduced specific surface area offsets any improvements in electronic and ionic transfer. In contrast, constructing low‐tortuosity, long‐range pore channels through directional freeze‐drying substantially enhances ion transport, and integrating these channels with CNT conductive networks enables simultaneous optimization of electron and ion transport. As a result, the optimized high‐mass‐loading cathode (10 mg cm^−2^) enabled a practical ZIHC pouch cell with a low N/P ratio (∼ 16.4), delivering 38.3 mAh g^−1^ at 10 A g^−1^ over 3000 cycles with a device‐level energy density of 2.82 Wh kg^−1^. More importantly, this work establishes a general design principle for thick carbon electrodes: improving electronic conductivity alone or introducing short‐range mesopores is insufficient to overcome transport limitations at practical mass loadings. Instead, high‐performance thick electrodes require the synergistic integration of low‐tortuosity, long‐range ion‐transport pathways with efficient electronic‐conductive networks. These findings provide practical guidelines for designing scalable carbon electrodes for ZIHCs and other electrochemical energy storage systems that employ high‐mass‐loading porous electrodes.

## Author Contributions

Jiacheng Wu: conceptualization, methodology, formal analysis, investigation, writing – original draft. Yangyang Wang: methodology, formal analysis, investigation. Fangzhou Liu: methodology, formal analysis, investigation. Fangxin She: methodology, formal analysis, investigation. Justin Prabowo: methodology, formal analysis, investigation. Di Zhu: methodology, formal analysis, investigation. Xin Yang: methodology, formal analysis, investigation. Li Wei: methodology, formal analysis, writing – review and editing. Yuan Chen: conceptualization, methodology, formal analysis, writing – review and editing, supervision, project administration, funding acquisition.

## Conflicts of Interest

The authors declare no conflicts of interest.

## Supporting information




**Supporting File**: advs76823‐sup‐0001‐SuppMat.docx.

## Data Availability

The data that support the findings of this study are available from the corresponding author upon reasonable request.
