# Supplementary material for: Breaking the Kinetics–Loading Trade‐Off in Zinc‐Ion Hybrid Capacitors Through Synergistic Electron–Ion Transport Design
Source: Adv Sci (Weinh). 2026 Jul 27;13(59):e76823. doi: 10.1002/advs.76823 (PMC13403732; doi:10.1002/advs.76823)
Supplement: Supplementary file 1 [file ADVS-13-e76823-s001.docx]

**Supporting Information**

**Breaking the kinetics–loading trade-off in zinc-ion hybrid capacitors through synergistic electron–ion transport design**

Jiacheng Wu, Yangyang Wang, Fangzhou Liu, Fangxin She, Justin Prabowo, Di Zhu, Xin Yang, Li Wei, Yuan Chen*

School of Chemical and Biomolecular Engineering, The University of Sydney, Darlington, NSW South Wales, 2006, Australia

AUTHOR INFORMATION

Corresponding Author

* [yuan.chen@sydney.edu.au](mailto:yuan.chen@sydney.edu.au) (Y.C)

**Experimental Section**

**1. *Chemicals and Reagents.*** Carbon black (Super-P, TIMCAL), single-walled carbon nanotube (CNT) aqueous dispersion (0.4 wt%, TUBALL, diameter = 1.6±0.4 nm), zinc sulfate heptahydrate (ZnSO_4_·7H_2_O, 99%, Sigma-Aldrich, ACS reagent), and Ti foil (10 μm, SCI materials hub) were used as received. Carboxymethylcellulose sodium salt (CMC) (M_w_ ~ 250,000, Ds = 0.9, Sigma-Aldrich) was predissolved in deionized (DI) water and mixed for 12 h to prepare a 2.0 wt% CMC solution. Zn foil (Zn, 50 *μ*m, SCI materials hub) was polished with 3000-mesh sandpaper, then washed with 80% ethanol before use. Commercial mesoporous carbons (HPC10 and HPC12) were purchased from Guangdong Jinwei Carbon & Energy Technology Co., LTD. Microporous carbon (YP-50F) was purchased from Kuraray Co., Ltd.

**2. *Material Characterizations.*** The morphologies of carbon materials and cathodes were characterized by scanning electron microscopy (SEM, Ultraplus, Zeiss; SEM, Phenom XL G2). N_2_ adsorption/desorption isotherms were acquired using Quantachrome Autosorb IQ instrumentation at 77 K. The specific surface area (SSA) was analyzed by a Brunauer-Emmett-Teller (BET) method. The micropore and mesopore size distribution of carbon materials was analyzed by the Density-Functional-Theory (DFT) method. Raman spectra were collected by a Raman microscope (Renishaw inVia Reflex microscope) using a 532 nm laser. X-ray diffraction (XRD) patterns were obtained from an XRD diffractometer (X-Pert, Malvern PANalytical) using a Cu-Kα X-ray source (λ = 1.5406 Å). The surface compositions of materials were analyzed by X-ray photoelectron spectroscopy (XPS, Thermo Scientific K-Apha+) with an AI-Kα X-ray source (1486.6 eV).

The X-ray micro-computed tomography (micro-CT) was performed at the Australian Synchrotron (ANSTO) beamline (beamline number 24011) in Melbourne. A monochromatic X-ray beam (20.0 keV) coupled with a mono detector was employed, providing a field of view (FOV) of 0.83×0.70 mm^2^ with an effective pixel size of 0.325 μm. The sample was continuously rotated through 180° with an exposure time of 0.07 s. Micro-CT datasets were further reconstructed at the beamline with Dragonfly 3D.

**3. *Fabrication of Carbon Electrodes.*** The low-mass-loading (2.0 mg cm^-2^) carbon electrodes were fabricated by mixing microporous carbon (YP-50F), conductive carbon black, and CMC binder at a mass ratio of 8:1:1, followed by the doctor-blade coating method onto Ti foil, vacuum drying at 80 °C for 12 h, and punching into 12 mm diameter disks. The high-mass-loading (10 mg cm^-2^) electrodes were fabricated using the same procedure, with an additional pre-drying step at 50 °C for 30 min before vacuum drying. The resulting electrodes are denoted as micro C–L and micro C–H, respectively.

**4. *Preparation of micro C–H + x% CNT Electrodes.*** For carbon electrodes incorporated with CNT conductive additives, microporous carbon (YP-50F), carbon black, and CMC binder (at a mass ratio of 8:1:1) were mixed with CNT aqueous dispersions at different CNT weight compositions (0.5, 1.0, and 2.0 wt%) with respect to the total mixture mass. The subsequent electrode fabrication procedure follows the same procedure described above. The resulting electrodes are denoted as micro C–H + x% CNT electrodes, where x refers to 0.5, 1.0, or 2.0 wt.%, respectively.

**5. *Preparation of micro C–H + x% meso C-1 and micro C–H + x% meso C-2 Electrodes.*** The fabrication procedure follows the same procedure as above, except that YP-50F was replaced by different mass compositions of YP-50F and HPC10/HPC12. The resulting electrodes are denoted as micro C–H + x% meso C-1 and micro C–H + x% meso C-2, where *x* refers to the mass fraction of HPC10 (meso C-1) or HPC12 (meso C-2) in the electrode at 20, 40%.

**6. *Preparation of DFD-micro C–H and DFD-micro C–H + x% CNT Electrodes.*** To prepare the directional freeze-dried high-mass-loading electrodes, the resulting slurry, with or without CNT additives, was transferred onto a cooling copper plate placed in a high-density foam insulation cold sink filled with liquid N_2_. The slurry was then frozen for 10 min and subsequently freeze-dried in a laboratory freeze dryer (–60 °C, 0.02 mbar, 24 h; CHRIST Alpha 1-2 LSCbasic). The resulting electrode without CNTs is denoted as DFD-micro C–H*.* The resulting electrodes with CNTs are denoted as DFD-micro C–H + x% CNT electrodes (x refers to CNT mass loadings of 0.5, 1.0, and 2.0 wt.%).

**7. *Electrochemical Tests.*** Zinc-ion hybrid capacitors (ZIHCs) were first assembled in CR2032 coin cells using a carbon cathode, a 50 μm zinc foil anode, a glass fiber separator (GF/D, Whatman), and 120 μL of 2.0 M ZnSO_4_ aqueous solution as electrolyte. Cyclic voltammetry (CV) was conducted at scan rates of 10–100 mV s⁻¹, and electrochemical impedance spectroscopy (EIS) was recorded with an AC voltage of 10 mV amplitude over a frequency range of 2×10^-3^ Hz–100 kHz using an electrochemical workstation (Autolab PGSTAT302N, Metrohm). Galvanostatic charge–discharge (GCD) and rate performance tests were performed within 0.2–1.8 V on a battery testing system (LAND, Wuhan, China). Distribution of Relaxation Times (DRT) calculations were analyzed via DRTtools.^[1]^

**9. *Assembly of Pouch Cells.*** Pouch cells were assembled using 10 µm Zn foil as anode (2 × 2 cm^2^), carbon cathode (2 × 2 cm^2^, active material mass loading of 2 or 10 mg cm^-2^), 2.0 M ZnSO_4_ aqueous electrolyte (500 *μ*L), and an NKK-TF4035 cellulose separator. The pouch cells were sealed with aluminum-plastic films and rested for 4 h before electrochemical testing. The breakdown of weights of all component in Zn||DFD-micro C–H + 2.0% CNT pouch cells is listed in **Table S6**.

**10. *Calculations of Specific Capacity, Energy Density, Warburg Coefficients, and Zn^2+^ diffusion coefficient****s*. The gravimetric specific capacities (*C*, mAh g^-1^) were determined from discharge curves by the following formula (**Equation 1**):

$$C=\frac{I\times\Delta t\times{10}^{3}}{3.6 m}$$

(1)

where *I* (A) is the applied current, *Δt* (s) is the galvanostatic discharge time, and *m* is the mass of active carbon materials (mg), respectively.^[2]^

The cell-level energy density (Wh kg^-1^) was calculated following **Equation 2**:

$$E_{g,cell}=\frac{U\times Q_{cell}}{m_{cell}}$$

(2)

where *U* is the working voltage window (1.6 V), *Q* is the total capacity of the cell (mAh), and *m_cell_* is the total cell weight (g) (i.e., cathode, anode).

Warburg coefficient (σ, Ω s^-0.5^) and Zn^2+^ diffusion coefficient (*D*_Zn_^2+^, cm^2^ s^-1^) are determined from EIS plots by **Equation 3** and **Equation 4**:

$$\omega=2\pi f$$

$$Z^{'}=\sigma\omega^{-0.5}+R_{s}+R_{ct}$$

(3)

$$D_{Zn^{2+}}=\frac{R^{2}T^{2}}{2A^{2}C^{2}n^{4}F^{4}\sigma^{2}}$$

(4)

where ω is the angle frequency (rad s^-1^), *f* is the frequency in Hz, Z’ is real part of impedance (Ω), *R_s_* is the electrolyte resistance (Ω), *R_ct_* is the charge transfer resistance (Ω), *n* is the number of electron transfer of Zn ions (n = 2), *A* is electrode surface area (1.13 cm^2^), *R* is the ideal gas constant (8.314 J mol^−1^ K^−1^), *T* is Kelvin temperature (298 K), *C* is molar concentration of electrolyte (2 mol L^-1^), and *F* is the Faraday constant (96485 C mol^-1^), respectively.^[3]^

$$D_{Zn^{2+}}\left( GITT \right)=\frac{4L^{2}}{\pi\tau}(\frac{\Delta E_{s}}{\Delta E_{\tau}})$$

(5)

The Zn^2+^ diffusion coefficient was also calculated in parallel with intermittent titration technique (GITT) measurements. A current pulse was applied to the assembled cell for 60 min at a current density of 0.2 A g^-1^, followed by a 30 min relaxation period. *D*_Zn_^2+^ (GITT) can be obtained based on **Equation 5**, where *L* is the electrode thickness (cm), τ is the constant current pulse time (s), Δ*E*_s_ is the potential change under the current pulse (V), and Δ*E*_τ_ is the potential change (V) under the constant pulse excluding the IR drop.^[4]^

**11. *Micro-CT Porosity and Tortuosity Analysis.*** The porosity and tortuosity analysis was performed using Avizo. The micro-CT datasets were first analyzed using the iterative thresholding method **(Figure S11**) for porous volume rendering. Volume fraction module, as well as Centroid Path Tortuosity module, were further applied to obtain the porosity and tortuosity information via the YZ orientation (**Table S4**).^[5]^

**Figure S1.** Rate performance of micro C-L and micro C-H pouch cells at various current densities (0.2–20 A g^-1^).

**Figure S2.** (a) The fitted linear relationship of Z’ and ω^-0.5^ for micro C-H and micro C-H + x% CNT electrodes. (b) *D_Zn_^2+^* values of micro C-H and micro C-H + x% CNT electrodes.

**Figure S3.** Long-term cycling stability of micro C-H and micro C-H + 2.0% CNT at 10 A g^-1^.

**Figure S4.** SEM images of (a) YP50-F, (b) HPC-10, and (c) HPC-12.

**Figure S5.** XRD patterns of YP-50F, HPC10, and HPC12.

**Figure S6.** Raman spectra of YP-50F, HPC10, and HPC12.

**Figure S7.** XPS survey spectra of YP-50F, HPC10, and HPC12.

**Figure S8.** High-resolution C1s XPS spectra of YP-50F, HPC10, and HPC12.

**Figure S9.** High-resolution O1s XPS spectra of YP-50F, HPC10, and HPC12.

**Figure S10.** $D_{Zn^{2+}}$ values of (a) micro C-H + x% meso-1 and (b) micro C-H + x% meso-2 electrodes.

**Figure S11.** (a-b) 2D cross-sectional micro CT slices of the (a) micro C–H + 40% meso C-1, (b) micro C–H + 40% meso C-2. (c-d) 3D micro CT volume rendering of the reconstructed (c) micro C-H + 40% meso C-1, (d) micro C-H + 40% meso C-2.

**Figure S12.** (a–d) 2D Region of interest (ROI, highlighted in blue) with iterative thresholding analysis of (a) micro C-H, (b) micro C-H + 40% meso C-1, (c) micro C-H + 40% meso C-2, and (d) DFD micro C-H. (e–h) Corresponding 3D volume renderings based on iterative thresholding for (e) micro C-H, (f) micro C-H + 40% meso C-1, (g) micro C-H + 40% meso C-2, and (h) DFD micro C-H.

**Figure S13.** (a) A photo of carbon materials coated on Ti film by the doctor-blade coating method. (b) Schematic diagram of the freezing device. (c) Photos of the freeze-drying process of the fabrication of carbon electrodes.

**Figure S14.** The thickness measurements of the DFD micro C-H electrode (a) before (~634 µm) and (b) after the cell assembly (~330 µm).

**Figure S15.** DRT calculated from *in-situ* EIS measurements at different potentials (0.2-1.8 V). (a) micro C-H electrode during the charge process. (b) micro C-H electrode during the discharge process. (c) DFD-micro C-H electrode during the charge process. (d) DFD-micro C-H electrode during the discharge process.

**Figure S16.** SEM images of top, bottom, and side views of (a-c) DFD-micro C–H + 0.5% CNT, (d-f) DFD-micro C–H + 1.0% CNT, and (g-l) DFD-micro C–H + 2.0% CNT.

**Figure S17.** (a) The fitted linear relationship of Z’ and ω^-0.5^ for DFD-micro C-H and DFD-micro C-H + x% CNT electrodes. (b) $D_{Zn^{2+}}$ values of DFD-micro C-H and DFD-micro C-H + x% CNT electrodes.

**Table S1.** The summarised *R_s_* and *R_ct_* values for 10 mg cm^-2^ micro C–H + x% CNT and DFD-micro C–H + x% CNT electrodes.

|  | ***R_s_* (ohm)** | ***R_ct_* (ohm)** |
| --- | --- | --- |
| **CNT** |  | |
| micro C–H | 1.38 | 68.5 |
| micro C–H + 0.5% CNT | 1.21 | 43.7 |
| micro C–H + 1.0% CNT | 1.13 | 36.6 |
| micro C–H + 2.0% CNT | 0.98 | 30.7 |

**Table S2.** Surface element compositions (at.%) of YP-50F, HPC-10, and HPC-12 determined by XPS.

|  | **Atomic Compositions (at.%)** | |
| --- | --- | --- |
|  | **C** | **O** |
| **YP-50F** | 91.56 | 8.44 |
| **HPC10** | 92.95 | 7.05 |
| **HPC12** | 92.28 | 7.72 |

**Table S3.** The summarised *R_s_* and *R_ct_* values for 10 mg cm^-2^ carbon composite electrodes with different HPC10 and HPC12 compositions.

|  | ***R_s_* (ohm)** | ***R_ct_* (ohm)** |
| --- | --- | --- |
| **HPC10** |  | |
| micro C–H | 1.38 | 68.5 |
| micro C–H+20% meso C-1 | 1.12 | 59.6 |
| micro C–H+40% meso C-1 | 1.12 | 49.5 |
| **HPC12** |  |  |
| micro C–H+20% meso C-2 | 1.17 | 47.7 |
| micro C–H+40% meso C-2 | 1.14 | 32.6 |

**Table S4**. The porosity and tortuosity analysis of micro C-H, micro C-H + 40% meso-1, micro C-H + 40% meso C-2, and DFD-micro C-H electrodes.

| **Sample** | **Volume Fraction** | **Label Volume** | | **Total Volume** | **Label Voxel Count** | **Total Voxel Count** | **Tortuosity** |
| --- | --- | --- | --- | --- | --- | --- | --- |
| micro C-H | 0.126525 | | 2.86E+07 | 2.26E+08 | 28643356 | 226385250 | 2.57502 |
| micro C-H + 40% meso C-1 | 0.103957 | | 2.58E+07 | 2.48E+08 | 25814060 | 248314900 | 3.1143 |
| micro C-H + 40% meso C-2 | 0.143831 | | 3.85E+07 | 2.68E+08 | 38496869 | 267653010 | 2.60989 |
| DFD-micro C-H | 0.832241 | | 5.91E+17 | 7.11E+17 | 591479394 | 710707008 | 1.34056 |

**Table S5.** The summarised *R_s_* and *R_ct_* values for 10 mg cm^-2^ micro C–H + x% CNT and DFD-micro C–H + x% CNT electrodes.

|  | ***R_s_* (ohm)** | ***R_ct_* (ohm)** |
| --- | --- | --- |
| **FZ-CNT** |  |  |
| DFD-micro C–H | 1.49 | 49.6 |
| DFD-micro C–H + 0.5% CNT | 1.38 | 36.7 |
| DFD-micro C–H + 1.0% CNT | 1.75 | 29.2 |
| DFD-micro C–H + 2.0% CNT | 1.55 | 26.7 |

**Table S6**. The breakdown of the weights of all components in Zn||DFD-micro C–H + 2.0% CNT pouch cell.

| **Component** | **Weight (mg)** |
| --- | --- |
| DFD-micro C–H + 2.0% CNT | ~40 |
| Zn foil anode (2×2 cm^2^) | 28.6 |
| NKK separator (2×2 cm^2^) | 22.4 |
| 2M ZnSO_4_ electrolyte (500 µL) | 655 |
| Total | 746 |

**References cited in the Supporting Information**

[1]	Z. Wang, Y. Wang, B. Py, A. Maradesa, J. Liu, T. H. Wan, M. Saccoccio, F. Ciucci, *ACS Electrochemistry* **2025**, *1*, 2680-2689. <https://doi.org/10.1021/acselectrochem.5c00334>.

[2]	J. Yin, W. Zhang, W. Wang, N. A. Alhebshi, N. Salah, H. N. Alshareef, *Adv. Energy Mater.* **2020**, *10*, 2001705. <https://doi.org/https://doi.org/10.1002/aenm.202001705>.

[3]	J. Huo, X. Wang, Z. Li, L. Zhang, G. Yue, S. Guo, *J. Energy Storage* **2025**, *107*, 115020. <https://doi.org/https://doi.org/10.1016/j.est.2024.115020>.

[4]	Q. Song, L. Jiang, H. Chen, H. Li, Y. Yang, S. Huang, L. Luo, Y. Chen, *Energy Storage Mater.* **2025**, *77*, 104219. <https://doi.org/https://doi.org/10.1016/j.ensm.2025.104219>.

[5]	a)J. Fu, H. R. Thomas, C. Li, *Earth-Sci. Rev.* **2021**, *212*, 103439. <https://doi.org/https://doi.org/10.1016/j.earscirev.2020.103439>; b)B. Li, S. Wang, P. He, Z. Huang, B. Mai, X. Zhang, Y. Chen, Y. Wen, X. Ran, C. Han, I. Manke, K. Dong, M. Yan, *Chem. Eng. J.* **2025**, *511*, 161931. <https://doi.org/https://doi.org/10.1016/j.cej.2025.161931>.
